# Baseline Optical Coherence Tomography Features and Visual Outcomes After Anti-vascular Endothelial Growth Factor Therapy for Retinal Vein Occlusion-Associated Macular Edema: A Prospective Observational Study

**DOI:** 10.7759/cureus.117185

**Published:** 2026-09-29

**Authors:** Garima Mandloi, Ekta Thakur, Anshul Tiwari, Sneha Alaway, Ahtesham Ansari

**Affiliations:** 1 Ophthalmology, Gandhi Medical College, Bhopal, IND; 2 Ophthalmology, Civil Hospital Sihora, Jabalpur, IND

**Keywords:** anti-vegf, disorganization of retinal inner layers, ellipsoid zone, macular edema, optical coherence tomography, retinal vein occlusion, visual outcome

## Abstract

Background

Macular edema is a major cause of visual loss in retinal vein occlusion (RVO). Although anti-vascular endothelial growth factor (anti-VEGF) therapy reduces retinal thickening, visual recovery varies. The primary objective was to assess whether baseline optical coherence tomography (OCT) features were associated with visual acuity one month after the third anti-VEGF injection using a prespecified 6/18 threshold. Secondary objectives assessed demographic and clinical associations, while central macular thickness (CMT) reduction was evaluated exploratorily.

Methodology

This prospective observational study included 60 eyes of 60 patients with center-involving, RVO-associated macular edema treated between September 2022 and February 2024. Each eye received three intravitreal anti-VEGF injections, with assessment one month after each injection. A good outcome was defined as final visual acuity of 6/18 or better. Baseline OCT features included CMT, macular volume, hyperreflective foci, serous macular detachment, ellipsoid zone (EZ) and external limiting membrane (ELM) integrity, disorganization of the retinal inner layers (DRIL), foveal bulge, and ganglion cell layer (GCL) cystic spaces. Categorical variables were compared using Fisher’s exact or Fisher-Freeman-Halton exact tests, and continuous variables using the independent-samples t-test.

Results

Good visual outcome occurred in 21 (35.0%) eyes. Younger age, branch RVO, and non-ischemic disease were associated with good outcomes (p = 0.001, p = 0.003, and p < 0.001, respectively). Baseline and final CMT were lower in the good-outcome group (both p < 0.001). Higher baseline macular volume, EZ disruption, DRIL, and ELM disruption were associated with poor outcomes, whereas foveal bulge was associated with good outcomes (all p < 0.001). CMT reduction >100 µm was not associated with visual outcome (p = 0.778).

Conclusions

Several baseline OCT features were associated with final visual outcome, whereas CMT reduction was not. These findings are exploratory and do not establish independent predictive value or clinical utility. Larger studies with standardized treatment, multivariable adjustment, and external validation are needed.

## Introduction

Retinal vein occlusion (RVO) is a common retinal vascular disease and an important cause of vision loss, particularly when macular edema develops. Intravitreal anti-vascular endothelial growth factor (anti-VEGF) therapy is a well-established intervention that delivers meaningful anatomical and functional gains in a large proportion of treated eyes [1,2]. Even when the edema regresses, the extent of visual recovery differs from eye to eye. Because a reduction in retinal thickness does not reliably translate into improved vision, the degree of underlying vascular and structural damage is thought to shape the ultimate prognosis [3,4].

Spectral-domain optical coherence tomography (SD-OCT) resolves the retinal layers in fine detail. Parameters such as central macular thickness (CMT), macular volume, hyperreflective foci, serous macular detachment, ellipsoid zone (EZ) integrity, external limiting membrane (ELM) integrity, disorganization of the retinal inner layers (DRIL), and foveal contour have all been examined as markers of disease severity and visual prognosis in RVO-associated macular edema [5,6].

The primary objective of this study was to assess whether baseline OCT features were associated with visual acuity one month after the third intravitreal anti-VEGF injection in eyes with RVO-associated macular edema, using a prespecified visual acuity threshold of 6/18. Secondary objectives were to assess the associations of baseline demographic and clinical characteristics with visual outcomes. An exploratory objective was to examine whether the magnitude of CMT reduction was associated with visual outcomes. Therefore, this study evaluated visual outcomes after intravitreal anti-VEGF therapy and assessed the baseline demographic, clinical, and OCT characteristics associated with visual outcomes in eyes with RVO-related macular edema.

## Materials and methods

Study design and setting

This prospective observational study was conducted in the Department of Ophthalmology, Shyam Shah Medical College, Rewa, Madhya Pradesh, India, between September 2022 and February 2024. In total, 60 eyes from 60 patients with center-involving macular edema secondary to RVO were enrolled.

Ethical Approval and Consent

The Institutional Ethics Committee of Shyam Shah Medical College, Rewa, approved the study (approval number: IEC/MC/2022/20909 dated September 12, 2022). All participants provided written informed consent before enrollment, and the study was conducted in accordance with institutional requirements for research involving human participants and with the principles of the Declaration of Helsinki.

Eligibility Criteria

The inclusion and exclusion criteria are summarized in Table 1. The flow of eyes through the study is summarized in Figure 1.

**Table 1:** Inclusion and exclusion criteria applied in the study. OCT: optical coherence tomography; RVO: retinal vein occlusion

Criterion type	Criterion
Inclusion	Center-involving macular edema secondary to RVO
	Central macular thickness greater than 250 µm on OCT
	OCT images of adequate quality at baseline and follow-up
Exclusion	Vitreoretinal conditions that could alter macular structure or visual acuity, such as age-related macular degeneration, uveitis, epiretinal membrane, vitreomacular traction, retinal detachment, or recent vitrectomy
	Glaucoma or other disorders affecting retinal thickness
	Intraocular surgery, retinal laser photocoagulation, or intravitreal injection within the preceding six months
	Poor OCT image quality

**Figure 1:**
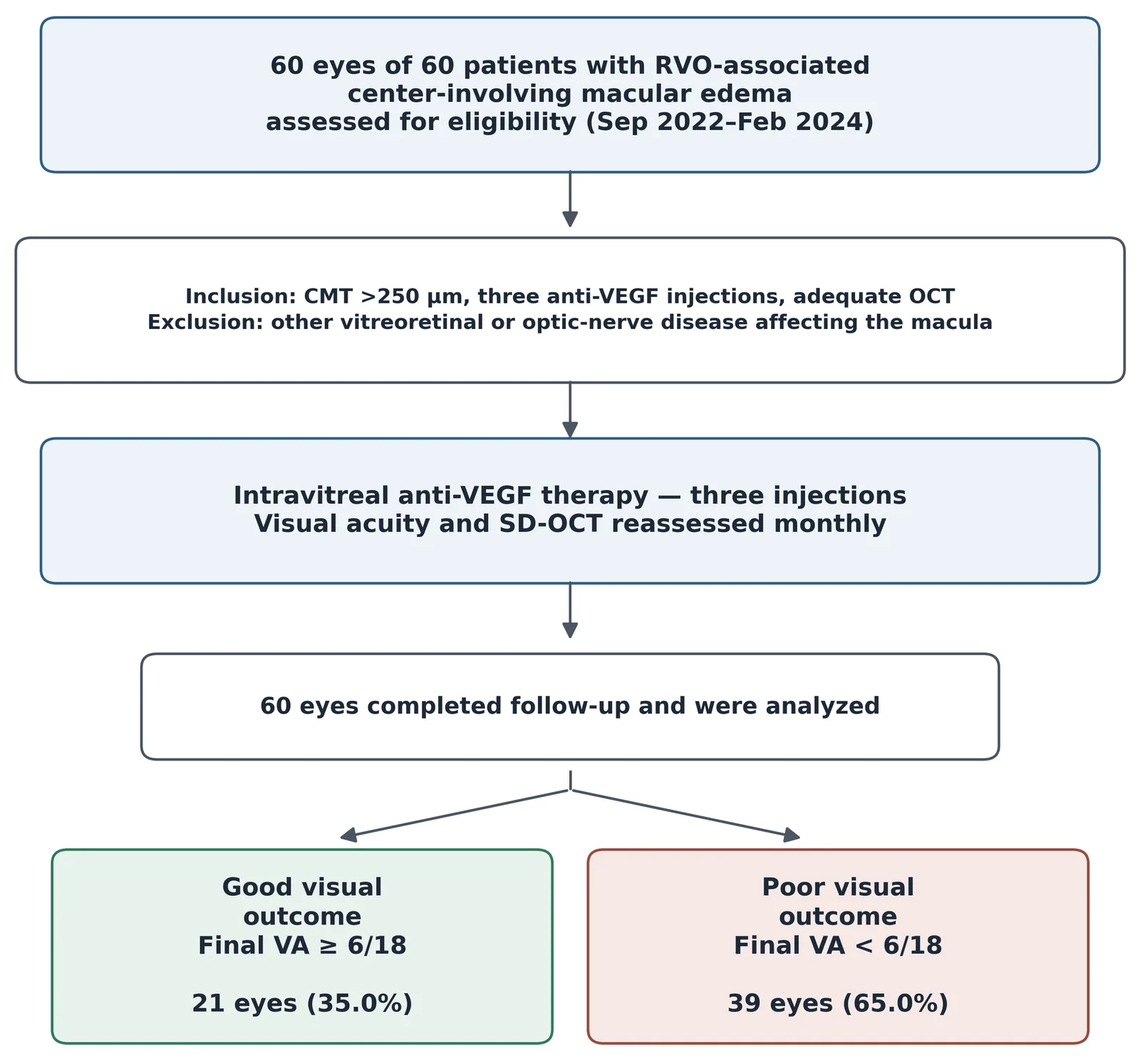
Flow of eyes through the study, from eligibility assessment to classification by visual outcome. RVO: retinal vein occlusion; VA: visual acuity; CMT: central macular thickness; OCT: optical coherence tomography; SD-OCT: spectral-domain optical coherence tomography; anti-VEGF: anti-vascular endothelial growth factor

Clinical Assessment and Classification of Retinal Vein Occlusion

Ocular and systemic histories were recorded for each patient. Best-available visual acuity was measured with a Snellen chart at 6 m. Intraocular pressure measurement, slit-lamp biomicroscopy, and stereoscopic fundus examination were performed. RVO was categorized as central retinal vein occlusion (CRVO), hemi-central retinal vein occlusion (HCRVO), or branch retinal vein occlusion (BRVO). Ischemic status was classified clinically, with fundus fluorescein angiography performed when perfusion status was uncertain. Ischemic RVO was defined by clinical features indicating retinal ischemia, extensive retinal hemorrhages, multiple cotton-wool spots, a relative afferent pupillary defect, or optic disc edema, and eyes without such features were classified as non-ischemic. The duration between RVO onset and initiation of treatment was not reliably available for all participants and was therefore not included in the comparative analysis. Ischemic status was classified using the available clinical and imaging information. A uniform angiographic threshold for ischemia was not consistently documented in the original records and therefore could not be retrospectively reconstructed.

Optical Coherence Tomography Assessment

Macular SD-OCT was acquired with the Cirrus OCT platform (Carl Zeiss Meditec, Dublin, CA, USA) using a 512 × 128 macular cube together with high-definition raster scans. Macular edema was defined as intraretinal or subretinal fluid accompanied by a CMT above 250 µm. The OCT variables assessed included CMT, macular volume, serous macular detachment, hyperreflective foci, EZ disruption, DRIL, foveal bulge, ganglion cell layer (GCL) cystic spaces, and ELM disruption. Visual acuity and OCT were repeated one month after each intravitreal injection.

Optical Coherence Tomography Biomarker Definitions and Assessment

On baseline macular SD-OCT scans, the following qualitative biomarkers were assessed as present or absent based on their characteristic OCT appearance: EZ disruption, ELM disruption, DRIL, foveal bulge, hyperreflective foci, serous macular detachment, and GCL cystic spaces. For exploratory analysis, baseline macular volume was categorized at a threshold of 11 mm³. The original study records did not consistently document the number of graders, detailed operational grading criteria, or intergrader agreement for these qualitative OCT features; therefore, these aspects could not be fully reconstructed retrospectively.

Treatment and Follow-Up

All included eyes received three intravitreal anti-VEGF injections at approximately four-week intervals. The anti-VEGF agent and dose were recorded when available in the clinical records. However, this information was not consistently available for all participants and therefore was not included as a comparative treatment variable in the present analysis. The interval between symptom onset and initiation of anti-VEGF therapy was not consistently documented and therefore could not be reliably analyzed. Visual acuity and OCT were assessed at baseline and approximately one month after each of the three intravitreal injections. The final visual outcome was assessed one month after the third intravitreal injection, corresponding to approximately three months after baseline.

Outcome Definitions

The primary endpoint was visual outcome at the final visit. Good visual outcome was defined as a final visual acuity of 6/18 or better; eyes with a final visual acuity worse than 6/18 were classified as having a poor visual outcome. The primary outcome was visual outcome assessed one month after the third intravitreal anti-VEGF injection. A reduction in CMT of more than 100 µm between baseline and the final assessment was defined as the anatomical response threshold.

Statistical analysis

Data were analyzed using SPSS version 20.0 (IBM Corp., Armonk, NY, USA). Categorical variables are presented as frequencies and percentages and were compared using Fisher’s exact test or the Fisher-Freeman-Halton exact test, as appropriate. Continuous variables are presented as mean ± standard deviation and were compared between the good- and poor-outcome groups using independent-samples t-tests, with Welch’s t-test used when equal variances could not be assumed. Effect sizes were reported as the phi coefficient (φ) for 2 × 2 comparisons, Cramér’s V for larger contingency tables, and Cohen’s d for continuous comparisons. Because several OCT biomarkers showed complete separation (no good outcomes among eyes with EZ disruption, DRIL, or ELM disruption) and the number of favorable outcomes was limited, conventional multivariable logistic regression was considered statistically unstable and was not performed; therefore, the reported associations were unadjusted and should not be interpreted as independent predictors or causal relationships. Because multiple OCT biomarkers were evaluated in this exploratory analysis, p-values were not adjusted for multiple comparisons and should be interpreted as hypothesis-generating. The primary analysis evaluated associations between baseline OCT features and visual outcome at one month after the third anti-VEGF injection. Demographic and clinical characteristics were evaluated as secondary analyses, while the association between the magnitude of CMT reduction and visual outcome was considered exploratory. A formal sample size calculation was not performed because all eligible patients presenting during the predefined study period were consecutively enrolled. A two-sided p-value below 0.05 was considered statistically significant.

## Results

Demographic and clinical associations

In secondary analyses, demographic and clinical characteristics were examined in relation to visual outcome. In total, 60 eyes from 60 patients met the eligibility criteria and were included in the study; all 60 completed the planned three-injection protocol and final assessment. A good visual outcome was recorded in 21 (35.0%) eyes and a poor outcome in 39 (65.0%) eyes. Baseline demographic and clinical characteristics according to visual outcome are summarized in Table 2. Patients with a good visual outcome were younger than those with a poor outcome. BRVO and non-ischemic disease occurred more often among good-outcome eyes, whereas sex showed no association with outcome.

**Table 2:** Demographic and clinical factors associated with visual outcome. Values are n (%) unless otherwise indicated. *: Fisher exact test; †: Fisher-Freeman-Halton exact test; age compared with the independent-samples t-test. Effect sizes: φ, phi coefficient; V, Cramér’s V; d, Cohen’s d. CRVO: central retinal vein occlusion; BRVO: branch retinal vein occlusion; HCRVO: hemi-central retinal vein occlusion; SD: standard deviation

Variable	Category	Good outcome (n = 21)	Poor outcome (n = 39)	P-value (effect size)
Age, years	Mean ± SD	51.85 ± 10.71	62.15 ± 10.94	0.001 (d = 0.95)
Sex	Male	10 (47.6)	19 (48.7)	1.000* (φ = 0.01)
	Female	11 (52.4)	20 (51.3)	
Type of RVO	CRVO	7 (33.3)	27 (69.2)	0.003† (V = 0.45)
	BRVO	13 (61.9)	7 (17.9)	
	HCRVO	1 (4.8)	5 (12.8)	
Nature of RVO	Non-ischemic	21 (100.0)	21 (53.8)	<0.001* (φ = 0.48)
	Ischemic	0 (0.0)	18 (46.2)	

Central Macular Thickness and Macular Volume

Baseline and final CMT were both lower in eyes with a good outcome. A baseline macular volume >11 mm³ was associated with a poor outcome (Table 3). The differences in baseline and final CMT between the good- and poor-outcome groups are illustrated in Figure 2A.

**Table 3:** Central macular thickness and baseline macular volume in relation to visual outcome. *: Fisher exact test; CMT values compared with the independent-samples t-test. Effect sizes: φ, phi coefficient; d, Cohen’s d. CMT: central macular thickness; OCT: optical coherence tomography; SD: standard deviation

OCT parameter	Category	Good outcome (n = 21)	Poor outcome (n = 39)	P-value (effect size)
Baseline CMT (µm)	Mean ± SD	434.85 ± 78.64	523.66 ± 104.08	<0.001 (d = 0.92)
Final CMT (µm)	Mean ± SD	281.52 ± 15.23	363.94 ± 92.41	<0.001 (d = 1.09)
Baseline macular volume	>11 mm³	13	38	<0.001* (φ = 0.48)
	≤11 mm³	8	1	

**Figure 2:**
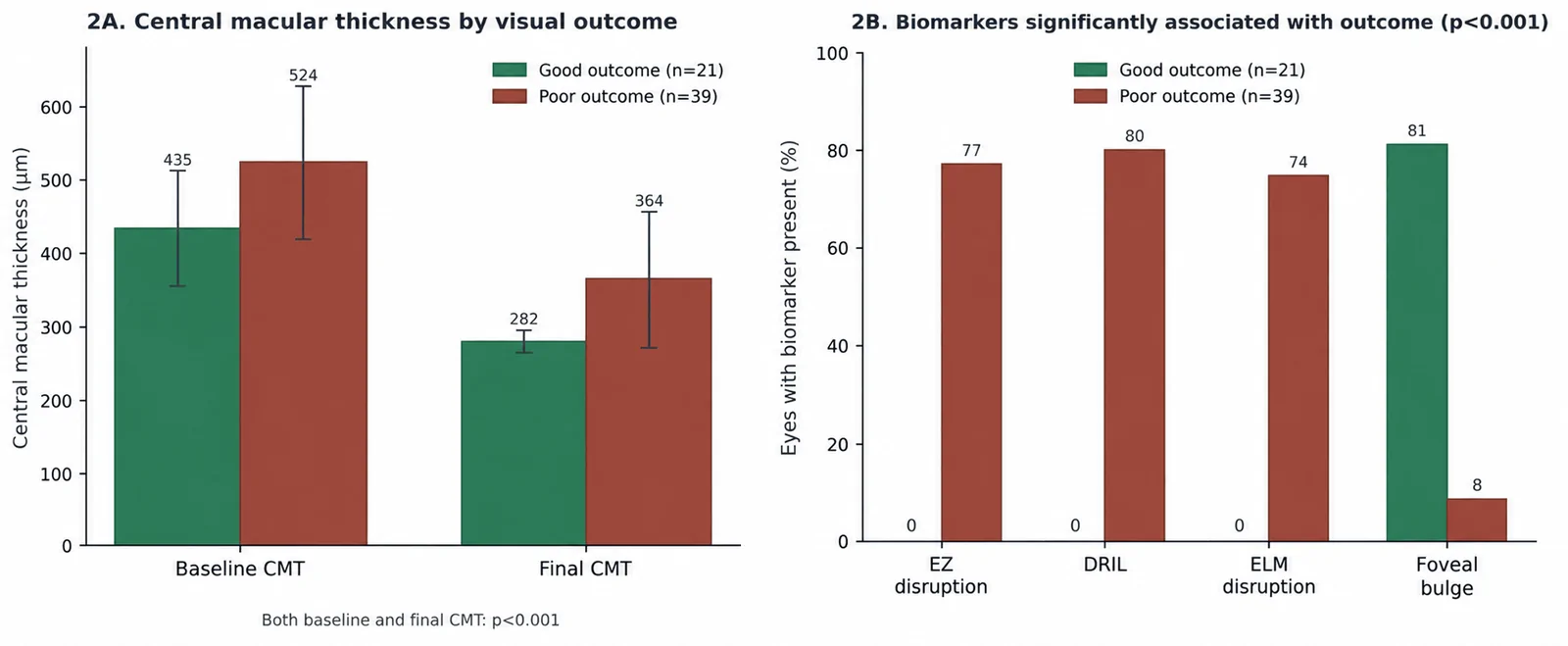
Key quantitative and qualitative OCT findings by visual outcome group. (A) Baseline and final CMT in the good- and poor-outcome groups; bars show the mean, and error bars show the standard deviation (both comparisons, p < 0.001). (B) Proportion of eyes showing each of the four biomarkers that were significantly associated with outcome, i.e., EZ disruption, DRIL, ELM disruption, and foveal bulge, by outcome group. The p-values for all evaluated biomarkers are presented in Table 3. CMT: central macular thickness; EZ: ellipsoid zone; DRIL: disorganization of the retinal inner layers; ELM: external limiting membrane

Qualitative Optical Coherence Tomography Biomarkers

EZ disruption, DRIL, and ELM disruption were observed predominantly in the poor-outcome group, whereas a foveal bulge was associated with a good outcome. Hyperreflective foci, serous macular detachment, and GCL cystic spaces were not significantly associated with visual outcome (Table 4). The proportions of eyes showing the four significantly associated OCT biomarkers are illustrated in Figure 2B.

**Table 4:** Baseline qualitative OCT biomarkers associated with visual outcome. *: Fisher exact test. Effect sizes expressed as the phi coefficient (φ). DRIL: disorganization of the retinal inner layers; ELM: external limiting membrane; GCL: ganglion cell layer; OCT: optical coherence tomography

Baseline OCT biomarker	Status	Good outcome (n = 21)	Poor outcome (n = 39)	P-value (effect size)
Hyperreflective foci	Present	2	8	0.470* (φ = 0.14)
	Absent	19	31	
Serous macular detachment	Present	7	8	0.352* (φ = 0.14)
	Absent	14	31	
Ellipsoid zone disruption	Present	0	30	<0.001* (φ = 0.73)
	Absent	21	9	
DRIL	Present	0	31	<0.001* (φ = 0.76)
	Absent	21	8	
ELM disruption	Present	0	29	<0.001* (φ = 0.71)
	Absent	21	10	
Foveal bulge	Present	17	3	<0.001* (φ = 0.74)
	Absent	4	36	
GCL cystic spaces	Present	4	8	1.000* (φ = 0.02)
	Absent	17	31	

Magnitude of Central Macular Thickness Reduction

A CMT reduction greater than 100 µm was observed in 41 of 60 (68.3%) eyes; however, the proportion of eyes achieving a good visual outcome did not differ according to the magnitude of CMT reduction (Table 5).

**Table 5:** Association between reduction in central macular thickness and visual outcome. *: Fisher exact test. Effect size expressed as the phi coefficient (φ). CMT: central macular thickness

CMT reduction after treatment	Good outcome (n = 21)	Poor outcome (n = 39)	Total, n (%)	P-value (effect size)
>100 µm	15	26	41 (68.3)	0.778* (φ = 0.05)
≤100 µm	6	13	19 (31.7)	

## Discussion

This prospective study assessed the clinical and baseline OCT features associated with visual outcome after intravitreal anti-VEGF therapy for RVO-associated macular edema. Although a CMT reduction exceeding 100 µm was achieved in 68.3% of eyes, only 21 of 60 (35.0%) eyes met the criterion for a good visual outcome. This gap reinforces the view that anatomical improvement by itself does not dictate functional recovery after treatment [3,4].

Age and RVO subtype were associated with visual outcomes. On average, patients with a good outcome were roughly a decade younger than those with a poor outcome, echoing earlier reports that younger age predicts better visual recovery after anti-VEGF therapy for RVO [7]. A favorable outcome was more common in BRVO, whereas CRVO/HCRVO and ischemic disease clustered with poorer results. No ischemic eye achieved a good outcome in this cohort, consistent with the more extensive capillary non-perfusion and neuronal injury characteristic of ischemic RVO; delayed retinal perfusion, indexed by a prolonged arm-to-retina time, has likewise been tied to differing visual trajectories in CRVO [8].

Baseline structural severity was also associated with visual outcome. Good-outcome eyes had lower baseline and final CMT, and a baseline macular volume > 11 mm³ was strongly associated with a poor outcome. Yet, the magnitude of the CMT reduction itself bore no relationship to visual outcome. This dissociation between anatomical and functional response has been described previously in anti-VEGF-treated RVO and argues that retinal thickness should be interpreted alongside microstructural integrity rather than as a stand-alone prognostic index [3,9].

The strongest associations involved outer and inner retinal integrity. EZ disruption, DRIL, and ELM disruption each aligned with a poor visual outcome, whereas a foveal bulge aligned with a favorable one. These relationships are biologically plausible: the EZ and ELM reflect photoreceptor and outer retinal integrity, while DRIL reflects disturbed inner retinal architecture and may signal broader neuronal damage. Comparable links between EZ or ELM disruption or DRIL and worse visual outcomes after anti-VEGF therapy for RVO have been reported by others [6,10,11]. The association between a foveal bulge and good outcome may reflect relative preservation of foveal photoreceptor architecture. Baseline EZ disruption and DRIL have similarly been associated with poorer visual acuity outcomes in RVO and CRVO treated with anti-VEGF therapy [12,13]. The observed associations should be considered exploratory because the study was small, the analyses were unadjusted, and several OCT features showed complete separation between outcome groups.

Hyperreflective foci, serous macular detachment, and GCL cystic spaces were not significantly related to outcome in this cohort. These null results warrant caution, because the study was not powered to detect small subgroup effects and some features were present in comparatively few eyes. Larger datasets have similarly found that baseline OCT features relate to presenting vision while most treated eyes still gain acuity, reinforcing the need for confirmation in larger cohorts with standardized regimens [14]. The findings should be interpreted in the context of the small single-center cohort, mixed RVO subtypes, limitations in visual acuity and ischemia standardization, incomplete treatment details, short follow-up, and the absence of multivariable adjustment. Larger studies with standardized outcome assessment and multivariable analyses are needed to determine whether these OCT features provide independent prognostic information.

Limitations

This study has several limitations. It was conducted at a single center and included only 60 eyes, limiting precision and generalizability. CRVO, HCRVO, and BRVO were analyzed together despite their different natural histories. Ischemic status was primarily classified using clinical features, with fluorescein angiography performed when perfusion status was uncertain; because a uniform angiographic threshold was not consistently documented, some classification variability cannot be excluded. The duration between symptom onset or RVO onset and treatment initiation was not reliably available for all participants. The specific anti-VEGF agent and dose could not be reliably reconstructed for each participant, preventing agent-specific analysis.

Visual acuity was recorded as the best-available Snellen acuity, and standardized testing conditions or conversion procedures were not consistently documented. Baseline and final visual acuity were used to define outcome categories but were not analyzed as continuous logMAR variables, limiting quantitative assessment of the relationship between baseline and final acuity. Follow-up was limited to the short-term treatment period, restricting assessment of longer-term visual stability and recurrence.

Reproducibility of qualitative OCT assessment was also limited because detailed operational grading criteria, grader information, and intergrader agreement were not consistently documented in the original records. Several OCT biomarkers showed complete separation between outcome groups, and only 21 eyes achieved a good outcome, precluding stable conventional multivariable logistic regression; therefore, the reported associations were unadjusted. The absence of adjustment for baseline visual acuity, together with the small sample size and multiple comparisons, limits the ability to determine whether the observed OCT associations are independent of baseline visual status or other clinical factors. Because multiple OCT biomarkers were evaluated without adjustment for multiple comparisons, the strength of these associations should be interpreted cautiously and considered hypothesis-generating rather than evidence of established independent predictors. These methodological limitations may also limit exact replication of the assessment procedures.

Despite these limitations, the prospective design and systematic evaluation of multiple OCT features provide useful exploratory evidence regarding the relationship between baseline retinal microstructure and visual outcome after treatment.

## Conclusions

Several baseline OCT features were associated with final visual outcomes after anti-VEGF therapy in this cohort. These findings are exploratory and should not be interpreted as evidence of independent predictive value or clinical utility. Larger studies incorporating baseline visual acuity, multivariable adjustment, and external validation are needed to determine whether these OCT features provide independent prognostic information.
